# Selection of a potential diagnostic biomarker for HIV infection from a random library of non-biological synthetic peptoid oligomers

**DOI:** 10.1016/j.jim.2016.05.001

**Published:** 2016-08

**Authors:** Tricia L. Gearhart, Ronald C. Montelaro, Mark E. Schurdak, Chris D. Pilcher, Charles R. Rinaldo, Thomas Kodadek, Yongseok Park, Kazi Islam, Raymond Yurko, Ernesto T.A. Marques, Donald S. Burke

**Affiliations:** a Center for Vaccine Research, University of Pittsburgh, 3501 Fifth Ave, Pittsburgh, PA, 15261, United States; b Drug Discovery Institute, University of Pittsburgh, 3501 Fifth Ave, Pittsburgh, PA 15261, United States; c Center for AIDS Research, University of California, 1001 Potrero Ave, SFGH 80, San Francisco, CA 94110, United States; d Graduate School of Public Health, University of Pittsburgh, 130 DeSoto Street, Pittsburgh, PA, United States; e The Scripps Research Institute, 130 Scripps Way, Jupiter, FL 33458, United States; f Peptide Synthesis Facility, University of Pittsburgh, 300 Technology Drive, Pittsburgh, PA 15219, United States

**Keywords:** HIV, Diagnostic, Biomarker, Peptoid, Infectious disease, ELISA

## Abstract

Non-biological synthetic oligomers can serve as ligands for antibodies. We hypothesized that a random combinatorial library of synthetic poly-*N*-substituted glycine oligomers, or peptoids, could represent a random “shape library” in antigen space, and that some of these peptoids would be recognized by the antigen-binding pocket of disease-specific antibodies. We synthesized and screened a one bead one compound combinatorial library of peptoids, in which each bead displayed an 8-mer peptoid with ten possible different amines at each position (10^8^ theoretical variants). By screening one million peptoid/beads we found 112 (approximately 1 in 10,000) that preferentially bound immunoglobulins from human sera known to be positive for anti-HIV antibodies. Reactive peptoids were then re-synthesized and rigorously evaluated in plate-based ELISAs. Four peptoids showed very good, and one showed excellent, properties for establishing a sero-diagnosis of HIV. These results demonstrate the feasibility of constructing sero-diagnostic assays for infectious diseases from libraries of random molecular shapes. In this study we sought a proof-of-principle that we could identify a potential diagnostic antibody ligand biomarker for an infectious disease in a random combinatorial library of 100 million peptoids. We believe that this is the first evidence that it is possible to develop sero-diagnostic assays – for any infectious disease – based on screening random libraries of non-biological molecular shapes.

## 1 Introduction

Non-biological synthetic oligomers can serve as ligands for antibodies (Reddy et al., 2011, Raveendra et al., 2013). Poly-*N*-substituted glycines, in which side chains of differing composition are linked to an oligomeric backbone by nitrogen atoms, rather than alpha-carbons, are peptide-like but non-biological. Such “peptoid” oligomers are easy to synthesize, inexpensive, and chemically stable to conventional enzymatic degradation and temperature, making them attractive candidates for use in diagnostic assays. We hypothesized that a random combinatorial library of synthetic poly-*N*-substituted glycine oligomers could represent a random “shape library” in antigen space, and that some of these peptoids would be recognized by the antigen-binding pocket of disease-specific antibodies. If the combinatorial library of oligomers is designed to be random and not engineered to be similar to any known antigen or antigens, the shape library could encompass a wide range of linear and non-linear conformational epitopes, post-translationally modified antigens, and other novel antigens. We reasoned that if this approach could be used to identify a diagnostic biomarker for any one infectious disease, it may be possible to do so for all infectious diseases. HIV was selected as our target disease for identification of a peptoid-based diagnostic biomarker. HIV, the etiological agent causing the epidemic of Acquired Immunodeficiency Syndrome – AIDS, has infected at least 70 million people world-wide and caused > 25 million deaths. Diagnostic assays for detection of antibodies to HIV are well standardized and widely used. However, all current antibody-based diagnostic assays for HIV are based on virus-derived antigens. The experiments described here present a radical departure in that we sought synthetic oligomer antigens that are not derived from HIV proteins. We applied an “antigenically agnostic” screening approach to the search for HIV-1 specific diagnostic biomarkers. This approach has previously been used to identify antibody ligand biomarkers for autoimmune diseases such as Alzheimer's disease and other chronic diseases not thought to be of infectious origin and where no antigen targets were known (Reddy et al., 2011, Raveendra et al., 2013).

## 2 Materials and methods

### 2.1 Peptoid library construction

The construction of the random one bead one compound peptoid library has been previously described (Lam et al., 1991, Figliozzi et al., 1996, Alluri et al., 2003, Raveendra et al., 2013). We used an Nlys-Nlys-Nleu invariant linker in library construction. Our library consisted of 8 monomer peptoids that were randomly generated from a series of 10 monomers. These 10 monomers were Nlys (1,4-Diaminobutane), Nleu (Isobutylamine), Ntyr (4-Methoxybenzylamine), Nmba (*R*-(+)-alphaMethylbenzylamine), Nchma (Cyclohexanemethylamine), Naea (*N*-(2-Aminoethyl)acetamide), Napp (*N*-(3-Aminopropyl)-2-pyrrolidinone), Nala (Beta Alanine), Npip (Piperonylamine), and Neth (2-Ethoxyethaneamine). The best diagnostic peptoid (HIV-DxP-1) identified through screening and extensive validation had the sequence of Npip (Piperonylamine)- Nala (Beta Alanine)- Naea (*N*-(2-Aminoethyl)acetamide)- Nmba (*R*-(+)-alphaMethylbenzylamine)- Neth (2-Ethoxyethaneamine)- Npip (Piperonylamine)- Nlys (1,4-Diaminobutane)- Npip (Piperonylamine).

### 2.2 Serum sample panels

For library screening and initial ELISA testing, we used seronegative serum purchased from MP Biomedicals (#823183), in addition to panels of plasma samples from the Consortium for the Evaluation and Performance of HIV Incidence Assays (CEPHIA). This panel included 202 plasma samples where 9 were HIV seronegative, and the remaining samples were HIV seropositive from various well-defined times post-seroconversion. The panel also included samples from individuals undergoing antiretroviral therapy.

The samples for testing of the best diagnostic candidate (36 HIV positive and 36 HIV negative individual serum samples) and the blinded serum panel (162 HIV positive and 49 HIV negative individual serum samples) used for confirmation testing in the peptoid ELISA was assembled by Dr. Charles Rinaldo of the University of Pittsburgh, and founder of the Multicenter AIDS Cohort Study (MACS) site in Pittsburgh, PA (Kaslow et al., 1987). This serum panel has been extensively studied. Included in both of these panels were samples from individuals undergoing antiretroviral therapy.

A protocol (PRO12110514 dated 7 Jan, 2013) for this work was reviewed and approved by the University of Pittsburgh Institutional Review Board. Based on the information provided to the IRB, it was determined that the investigator conducting research would not obtain information about research subjects via an interaction with them, nor obtain identifiable private information.

### 2.3 Peptoid library screening and sequencing

The library screening procedure has been previously described (Raveendra et al., 2013). Briefly, the library was first screened with commercially available, seronegative serum; any peptoid/beads that bound antibodies present in normal serum were removed. We then screened the remaining beads with serum pools of 4 individual HIV negative serum samples from CEPHIA to completely denude the peptoid/bead library of any seronegative serum binding peptoids. The remaining library was screened with HIV positive serum pools of 4 individual samples from individuals 0–4 months post-seroconversion. Peptoid/beads that positively reacted were removed and saved for future study. This same process was repeated with the remaining peptoid/bead library with samples from 5 to 8 months post-seroconversion, 9–12 months post-seroconversion, 13–18 months post-seroconversion, 19–24 months post-seroconversion, and lastly long-term, antiretroviral-treated samples. All HIV positive samples in this panel were infected with Clade B. Human antibody binding to the peptoid/beads in these screening steps was determined by secondary binding of fluorescently labeled anti-human IgG, and visual screening by fluorescence microscopy. Any peptoid/beads that reacted positively with HIV positive serum were saved and retested in both pools and individual samples from the initial screening pool. Peptoid/beads that continued to react positively were sequenced as previously described using MS/MS mass spectrometry (Raveendra et al., 2013).

### 2.4 Peptoid ELISA

The ELISA assay that was used to quantitatively determine the seroreactivity of our potential peptoid biomarkers has been previously described (Ball et al., 1994). Briefly, we cross-linked the peptoids to the polystyrene plate surface of 96-well ELISA plates using poly-*l*-lysine as the anchor protein, and glutaraldehyde to covalently link the peptoids to the poly-*l*-lysine. 50 μg peptoid/well was immobilized on the plate surface. Wells were blocked in two steps, first with 1 M glycine, and then with 2.5% milk/0.5% gelatin (Sigma G2500). Primary human serum was added in the wells at a 1:50 dilution followed by washing the wells with 1 × phosphate buffered saline (PBS). Wells were then incubated in anti-human secondary antibody (Sigma A8792-2ML), washed again with 1 × PBS, and then reactivity was determined using TMBlue (Sigma T0440-1L) developing solution.

## 3 Results

We employed a one bead one peptoid compound library synthesis technique (Lam et al., 1991, Alluri et al., 2003, Raveendra et al., 2013). The peptoids contained eight variable positions, after an invariant three-residue linker. Ten different amines (selected to represent a range of chemical properties for size, hydrophobicity, and charge at each site) were used in a combinatorial synthesis approach, resulting in ten possible monomers at each of eight positions, for a theoretical diversity of 10^8^ distinct oligomers.

**Fig. 1:**
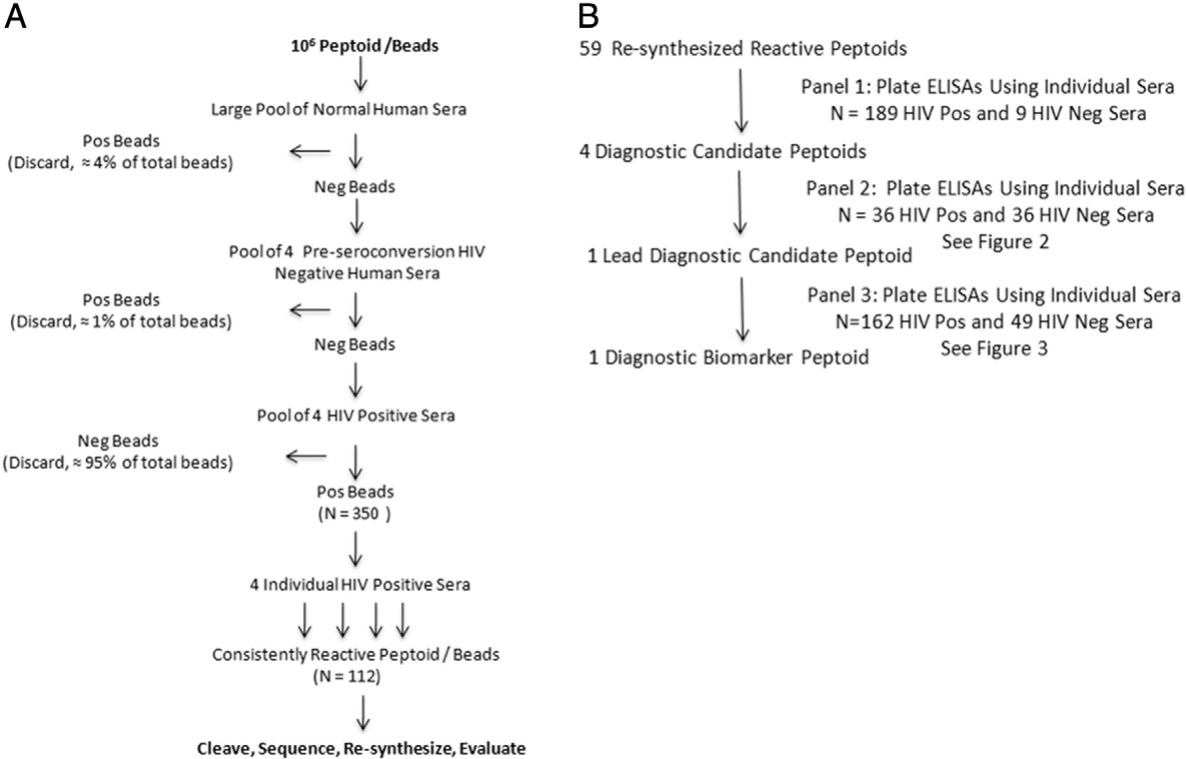
Peptoid Screening and Evaluation Flowcharts. a. Flowchart of peptoid library screening. b. Flowchart of reactive peptoid evaluation and diagnostic peptoid selection.

To identify peptoids that bind anti-HIV antibodies, the peptoid/bead library was screened with a step-wise series of well-characterized known HIV antibody-negative and HIV antibody-positive serum samples (Fig. 1a). Presence or absence of HIV antibody in these serum samples had previously been defined by testing with licensed HIV diagnostic assays. 50,000 beads were screened in each set, and a total of 1 million beads were screened. The library was first depleted of peptoid/beads that bound antibodies present in seronegative serum pools and known pre-infection HIV negative serum. Approximately 5% of the total peptoid/beads showed some measure of reactivity with seronegative and HIV negative human sera. The remaining peptoid/beads were incubated with pools of HIV positive serum from four to five individual patients. This Panel 1 of HIV positive serum samples included samples from individuals with both recent and long-standing infections, and individuals undergoing antiretroviral therapy. Any peptoid/beads that bound antibodies in these pools were removed and saved. For identification of peptoids, bound antibodies were eluted with SDS from the initially reactive peptoid/beads, and the candidate beads re-tested with each of the individual specimens of the HIV positive sera that had composed that positive pool. Through this iterative process, 112 peptoid/beads were detected that specifically and consistently bound antibodies present in all HIV positive serum but not seronegative human nor negative sera from persons who were known to later become infected by and sero-convert to HIV. Consistently reactive peptoids were cleaved from the beads and the peptoid sequences were determined using MS/MS mass spectrometry. Although the quantity of peptoid harvested from a single bead was sometimes insufficient for sequencing from some beads, and ambiguous sequences were obtained from others, a definitive sequence was obtained for a majority of the consistently reactive peptoids.

A total of 59 consistently reactive peptoids were re-synthesized in larger quantities for further testing. Synthesis was again done on beads, but unlike earlier steps in which every bead featured a unique combinatorial sequence, in this step all beads in each synthesis run shared the same sequence. Peptoids were cleaved and purified using HPLC. All 59 re-synthesized candidate peptoids were next tested in a plate ELISA format against the same set of individual serum samples that had been used to initially screen the bead/peptoid library (Panel 1, 189 HIV positive and 9 HIV negative sera; Fig. 1b). This 96-well microplate ELISA assay was modified from our previously published peptide ELISA protocol (Ball et al., 1994). In preliminary tests, simple adherence of the peptoids directly to the plate surface performed poorly, so we cross-linked the peptoids to the polystyrene plate surface using poly-*l*-lysine as the anchor protein, and glutaraldehyde to covalently link the peptoids to the poly-*l*-lysine. This procedure has been shown to significantly increase sensitivity as compared to ELISA assays where peptides were passively coated to the plates (Ball et al., 1994). In this assay, we demonstrated that the majority of the peptoids showed the expected reactivity patterns, with the quantitative optical density results in the plate ELISA format reproducing the qualitative results obtained in the visual bead screening format. Interestingly, different peptoids showed varying patterns and degrees of reactivity to various groups of serum samples. For the purpose of this study, we selected the peptoids that had the best diagnostic properties and reacted with all HIV positive serum in the ELISA format for more detailed study.

**Fig. 2:**
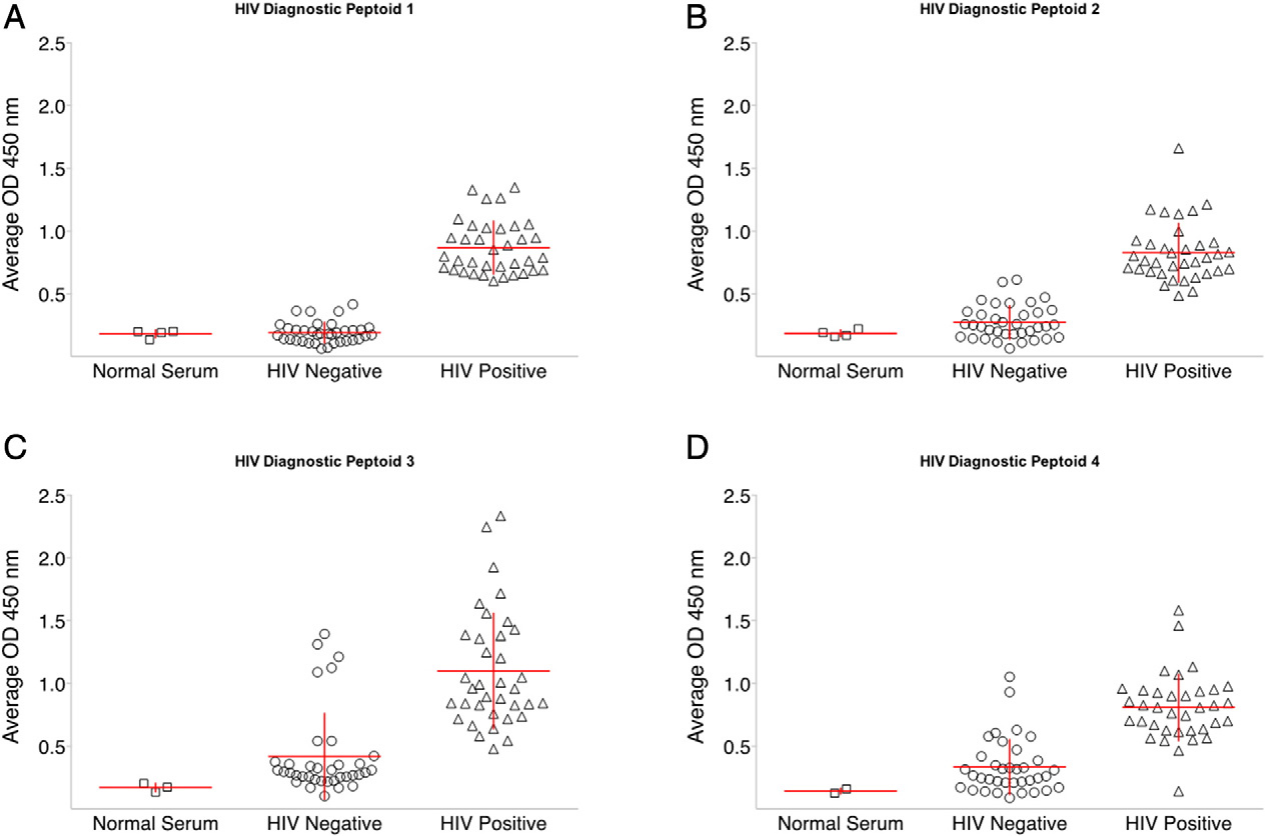
Diagnostic candidate peptoids can distinguish between individual HIV positive and HIV negative serum samples. a–d. Each point represents the average optical density of duplicate samples of an individual serum sample in the standard peptoid ELISA. Horizontal and vertical lines represent the mean and standard deviation respectively. With all four candidate peptoids in a–d, optical density values obtained were greater on assays using known HIV positive sera than with known HIV negative sera (p < 0.001, Student' *t*-test).

**Fig. 3:**
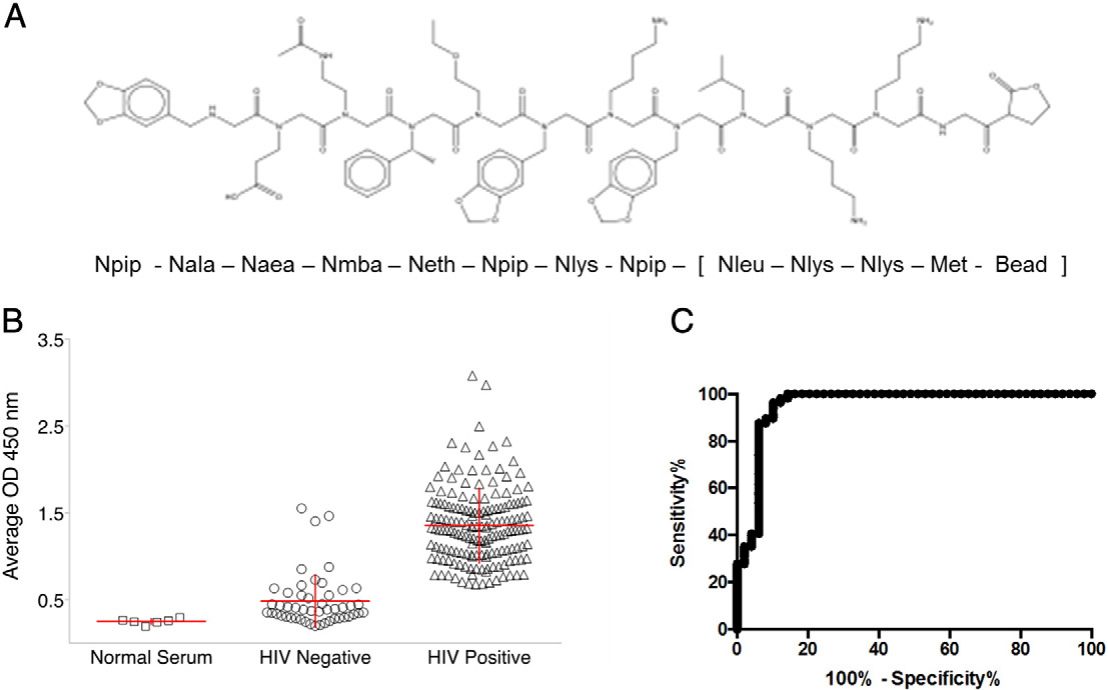
Analysis of potential diagnostic biomarker peptoid HIV-DxP-1: a. chemical structure and sequence of HIV-DxP-1 (see methods for chemical names of peptoid residues). b. Peptoid ELISA analysis of HIV-DxP-1 against 162 HIV positive and 49 HIV negative individual serum samples. Each symbol represents the average optical density of duplicate runs on an individual serum sample. Horizontal and vertical lines represent the means and standard deviations, respectively. Optical density values obtained were greater on assays using known HIV positive sera than with known HIV negative sera (p < 0.0001, Student' *t*-test). c. Receiver Operating Characteristic (ROC) curve of peptoid ELISA data showing sensitivity versus (100-specificity) for varying cut-off values.

The antibody binding properties of these four diagnostic candidate peptoids were examined using a second, independent panel of serum samples from patients in the Multicenter AIDS Cohort Study (Kaslow et al., 1987). This Panel 2 consisted of well-characterized serum samples from 36 HIV positive and 36 seronegative volunteers, all from the same epidemiological cohort (Fig. 2). While all four peptoids distinguished between HIV positive and seronegative samples (p < 0.001, Student's *t*-test), HIV-DxP-1 (Fig. 2a) showed the greatest separation between HIV positive and seronegative samples and was selected as the lead diagnostic candidate peptoid. We then tested HIV-DxP-1 (Fig. 3a) against a third set of unique serum samples. Panel 3 included 162 HIV positive and 49 seronegative volunteers from the Multicenter AIDS Cohort Study. Samples were coded and tested blindly. The unblinded results (Fig. 3b) demonstrated that the HIV-DxP-1 peptoid ELISA reliably distinguished between HIV positive sera and HIV negative sera (p < 0.0001, Student's *t*-test). The receiver operating characteristic (ROC) curve of the results (Fig. 3c) showed sensitivity and specificity values of approximately 90% or greater over a range of cut-off values. Because we have not yet fully optimized the HIV-DxP-1 ELISA assay conditions, we do not report precise sensitivity and specificity values here, but will do so as we further develop the assay.

## 4 Discussion

These results demonstrate that diagnostic antibody ligands for HIV were present in a random library of non-biological synthetic oligomers. Furthermore, we only had to screen 1 million peptoids in a library of 100 million theoretical variants to find at least one that showed excellent sensitivity and specificity properties. We believe that this is the first evidence that it is possible to develop diagnostic assays – for any infectious disease – based on screening random libraries of non-biological molecular shapes. There is no a priori reason to believe that our first peptoid library was especially tailored to generate HIV antibody ligands, so it is possible that other infectious diseases could be diagnosed with assays based on this or similar platforms. Additionally, future experiments are being conducted in order to explore the ability of peptoids to distinguish between recent and long-term HIV infections, an advance that would be important in the public health field.

One limitation of this approach is that we do not know the identity of the presumed natural ligand that corresponds to the non-natural HIV-DxP-1 peptoid. It could be a native HIV molecule, a denatured HIV molecule, or even a host-derived molecule. A technique for the determination of an unknown antigen and its corresponding antibody has previously been accomplished using a Type 1 Diabetes model (Doran et al., 2015). The natural ligand corresponding to HIV-DxP-1 will be determined in future studies.

Lastly, biologically inspired but non-natural antibody ligands such as HIV-DxP-1 may have value as immunogens. Here we screened our library for peptoids with “yes/no” diagnostic properties. By instead screening the library for ligands of neutralizing antibodies, or neutralizing monoclonal antibodies, it may be possible to discover protective immunogens.

## 5 Conclusions

In this work, we identified a novel potential biomarker for HIV-1 infection by screening a combinatorial library of synthetic, peptide-like, but nonbiological molecules called “peptoids”. This potential peptoid biomarker was able to distinguish between HIV-1 infect individuals and non-infected controls from the same cohort with high specificity and sensitivity. To our knowledge, this is the first evidence that peptoids can be used as biomarkers for diagnosing an infectious disease.

## Role of the funding source

The funder of the study had no role in study design, data collection, data analysis, data interpretation, or writing of the report.

## Declaration of interests

There are no conflicts of interest with this work.
